# Survey of Mental Health Professionals’ Knowledge and Skills in Managing Substance Misuse in Patients Admitted on a Mental Health High Dependency Unit

**DOI:** 10.1192/bjo.2025.10485

**Published:** 2025-06-20

**Authors:** Heba Salem, Nidhi Gupta

**Affiliations:** BSMHFT, Birmingham, United Kingdom

## Abstract

**Aims:** Dual diagnosis is very common in patients who have a psychotic disorder. This impacts symptom severity, treatment outcomes, and relapse rates. Following multiple incidents of drug misuse on a high dependency unit and inconsistent staff approach in dealing with these issues, we recognized the need to assess staff knowledge as a first step toward providing effective patient care. The aim of this survey was to assess knowledge and skills of mental health professionals to manage patients who misused substances while being admitted to a high dependency unit.

**Methods:** Data was collected using an online questionnaire to evaluate staff’s knowledge and perception of substance-related mental health risks with occasional and regular use and their role in managing it.

**Results:** 23 professionals participated in this survey – psychiatric nurses, healthcare assistants, occupational therapist, and psychologists. 72% of respondents believed occasional cannabis use while 90% believed regular use could exacerbate mental illness, 100% agreed that cannabis worsened existing mental health conditions with 95% feeling the need to counsel patients against its use.

In terms of class A drugs, 95% agreed that occasional use could cause mental health problems, while 100% recognized that these substances used long term could lead to worsening of mental illness.

86% were aware of the importance of drug and alcohol history on admission with 81% believed in providing advice and guidance. 91% supported referral to COMPASS (Specialist Drug Services). 78% felt they could diagnose opiate overdose and 100% were aware of naloxone use in opiate overdose. 100% recognized the importance of urine drug screening while 76% supported searches without consent if necessary. 55% felt police should be notified, and 45% supported placing patients on a contract, where discharge is part of the contract if breached.

91% agreed that staff required specialist training. Knowledge of synthetic opioids was limited, with only 53% recognizing their impact on mental health.

32% staff believed occasional alcohol use could worsen existing psychosis, while 77% recognized risks of heavy intake. 100% agreed that alcohol exacerbates existing mental illness.

**Conclusion:** Mental health professionals on HDU demonstrated awareness of substance-related mental health risks, particularly related to class A drugs, chronic cannabis and alcohol misuse. However, variations in patient management approaches, and lack of knowledge about synthetic opioids indicate the need for enhanced training.

